# Have You Heard of Schistosomiasis? Knowledge, Attitudes and Practices in Nampula Province, Mozambique

**DOI:** 10.1371/journal.pntd.0004504

**Published:** 2016-03-04

**Authors:** Christian Rassi, Dan Kajungu, Sandrine Martin, Jorge Arroz, Jamie Tallant, Celine Zegers de Beyl, Helen Counihan, James N. Newell, Anna Phillips, Jane Whitton, Artur Manuel Muloliwa, Kirstie Graham

**Affiliations:** 1 Malaria Consortium, London, United Kingdom; 2 Malaria Consortium, Kampala, Uganda; 3 Malaria Consortium, Maputo, Mozambique; 4 The Nuffield Centre for International Health & Development, University of Leeds, Leeds, United Kingdom; 5 Schistosomiasis Control Initiative, Imperial College, London, United Kingdom; 6 Direcção Provincial de Saúde de Nampula, Nampula, Mozambique; KEMRI, KENYA

## Abstract

**Background:**

Schistosomiasis is a parasitic disease which affects almost 300 million people worldwide each year. It is highly endemic in Mozambique. Prevention and control of schistosomiasis relies mainly on mass drug administration (MDA), as well as adoption of basic sanitation practices. Individual and community perceptions of schistosomiasis are likely to have a significant effect on prevention and control efforts. In order to establish a baseline to evaluate a community engagement intervention with a focus on schistosomiasis, a survey to determine knowledge, attitudes and practices relating to the disease was conducted.

**Methodology/Principal Findings:**

A representative cross-sectional household survey was carried out in four districts of Nampula province, Mozambique. Interviews were conducted in a total of 791 households, using a structured questionnaire. While awareness of schistosomiasis was high (91%), correct knowledge of how it is acquired (18%), transmitted (26%) and prevented (13%) was low among those who had heard of the disease. Misconceptions, such as the belief that schistosomiasis is transmitted through sexual contact (27%), were common. Only about a third of those who were aware of the disease stated that they practiced a protective behaviour and only a minority of those (39%) reported an effective behaviour. Despite several rounds of MDA for schistosomiasis in the recent past, only a small minority of households with children reported that at least one of them had received a drug to treat the disease (9%).

**Conclusion/Significance:**

Poor knowledge of the causes of schistosomiasis and how to prevent it, coupled with persisting misconceptions, continue to pose barriers to effective disease prevention and control. To achieve high levels of uptake of MDA and adoption of protective behaviours, it will be essential to engage individuals and communities, improving their understanding of the causes and symptoms of schistosomiasis, recommended prevention mechanisms and the rationale behind MDA.

## Author Summary

Schistosomiasis is one of the 17 ‘neglected tropical diseases’ as classified by the World Health Organization. It is a parasitic disease of poverty that affects around 300 million people each year, causing chronic ill-health and severe morbidity. The disease, which is spread through contact with freshwater contaminated with the parasite, can be prevented and controlled in two ways: by distributing drugs on a mass scale (“mass drug administration”) and by changing hygiene and sanitation practices to reduce contact with the parasite. A prerequisite for both strategies is that communities have an understanding of the disease and what solutions are available for its prevention and control.

In order to better understand knowledge, attitudes and practices surrounding schistosomiasis, Malaria Consortium carried out a survey in Nampula province, Mozambique, where the organisation is implementing a community engagement intervention with a focus on this disease. The findings indicate that while awareness of the disease itself is high, knowledge of how it is acquired, transmitted, prevented and treated is low. This suggests a need for greater efforts to improve knowledge of schistosomiasis in affected communities in order to increase uptake of mass drug administration and ensure that communities take appropriate measures to prevent infection.

## Introduction

Schistosomiasis is a parasitic disease acquired when people come into contact with larval forms of the parasite of the *Schistosoma* (*S*.) genus, known as cercariae, which are released by freshwater snails acting as intermediate hosts. In Africa, the main species infecting humans are *S*. *haematobium* and *S*. *mansoni*. Infection typically occurs while wading, bathing or washing in contaminated water, when cercariae emerging from their host snails penetrate a person’s skin and develop into mature worms, which can live within the blood vessels of the human host for several years. Acute symptoms after infection include fever, malaise, muscle pain and rashes. If left untreated, chronic disease, caused predominantly by the eggs from mated mature female worms passing through the body, can develop. For *S*. *mansoni* infections (intestinal schistosomiasis), this can include abdominal pain, diarrhoea, rectal bleeding and irreversible liver damage, and for *S*. *haematobium* infections (urogenital schistosomiasis), this can result in blood in urine, painful and frequent urination, bladder cancer and kidney failure. The complex life cycle of schistosomiasis continues when an infected person defecates or urinates in or near freshwater, releasing a proportion of the eggs into the water, which then hatch and infect their intermediate snail host [1].

Schistosomiasis is one of 17 neglected tropical diseases (NTDs) as classified by the World Health Organization (WHO). In 2013, there were an estimated 291 million cases of schistosomiasis worldwide [2]. WHO recommends preventive chemotherapy and transmission control (PCT) with praziquantel for the prevention, control and treatment of the disease [3], which is commonly provided to target populations through mass drug administration (MDA), i.e. the treatment of entire populations irrespective of disease status. To maximise populations’ protection, PCT needs to be complemented by effective case management, snail control and the adoption of basic hygiene and sanitation practices, such as adequate sanitation and water collection from safe water sources to avoid contact with the parasite [4].

Over 90% of all people who require PCT for schistosomiasis live in sub-Saharan Africa [5]. Mozambique is one of the worst affected countries, with an estimated 19 million of a total population of 23 million people requiring PCT for schistosomiasis in 2010 [6]. It is thought that 47% of school-age children in Mozambique are infected with the disease [7]. As one of the signatories of the London Declaration on NTDs, the Republic of Mozambique has committed to implementing a multi-sectoral plan to control and eliminate NTDs, targeting lymphatic filariasis, schistosomiasis and trachoma in endemic areas [8].

Individual and community perceptions, experiences and understanding of schistosomiasis are likely to have a significant effect on prevention and control efforts. A range of social and political factors such as not understanding the rationale of MDA, fear of side effects and lack of trust in drug distributors may influence individuals’ and communities’ willingness to participate in campaigns [9–11]. Community perceptions of the diseases will also influence how widely protective behaviours are adopted among populations living in endemic areas. For this reason, prevention and control interventions need to invest in social mobilisation and community participation [12–15], taking into account knowledge gaps, misconceptions and barriers to adoption of recommended behaviours. It is therefore important to gain a better understanding of knowledge, attitudes and practices (KAP) with regard to schistosomiasis at the community level.

Malaria Consortium, in partnership with the Republic of Mozambique’s Ministério de Saúde (Ministry of Health) and the Direcção Provincial de Saúde (Provincial Health Authority) in Nampula province, is conducting operational research to assess the effectiveness of the community dialogues approach to improve schistosomiasis prevention and control at community level. The community dialogue approach is a community engagement strategy to raise communities’ demand for and utilisation of health services and uptake of protective practices. It involves providing training on a health issue and basic facilitation techniques to non-specialist volunteers selected by the communities themselves. The volunteers are equipped with a set of visual tools designed to stimulate discussions among the community, as well as a guidebook that leads them through a simple, repeatable ten-step process for conducting regular community dialogues in their communities. The community dialogue approach prompts and enables communities to explore how a health issue affects their community, identify locally relevant solutions and plan for taking individual and communal action. It has been previously used by Malaria Consortium in the context of integrated community case management of childhood illnesses in Mozambique, Zambia and Uganda [16]. In collaboration with the Provincial Health Authority in Nampula province, Malaria Consortium trained approximately 160 community volunteers in four districts of Nampula province in August 2014. The volunteers conducted community dialogue sessions on the subject of schistosomiasis in their respective communities between September 2014 and November 2015.

In order to assess the effectiveness of the community dialogues approach in improving knowledge and understanding, adoption of recommended behaviours and community participation with regard to schistosomiasis prevention and control, the study involves a range of evaluation activities, including baseline and endline household KAP surveys. This manuscript summarises the findings from the baseline KAP survey conducted in June 2014, before community dialogues were implemented.

## Methods

### Study area

Nampula province has one of the highest prevalence rates of schistosomiasis in Mozambique. Average prevalence among school-age children is 78%, with a number of districts recording 90% prevalence among school-age children. The most common schistosome species in the region is *S*. *haematobium* [7]. The districts of Mecubúri, Eráti, Murrupula and Mogovolas were purposively selected as intervention districts in consultation with the Provincial Health Authority in Nampula province, as they are representative of the province, using the following criteria:

- High schistosomiasis prevalence rates.
- Socio-geographic conditions, with the majority of inhabitants practicing subsistence agriculture, growing rice or vegetables in or near riverbeds.
- Challenges with regard to schistosomiasis prevention and control, including a lack of access to clean water.

**Fig 1:**
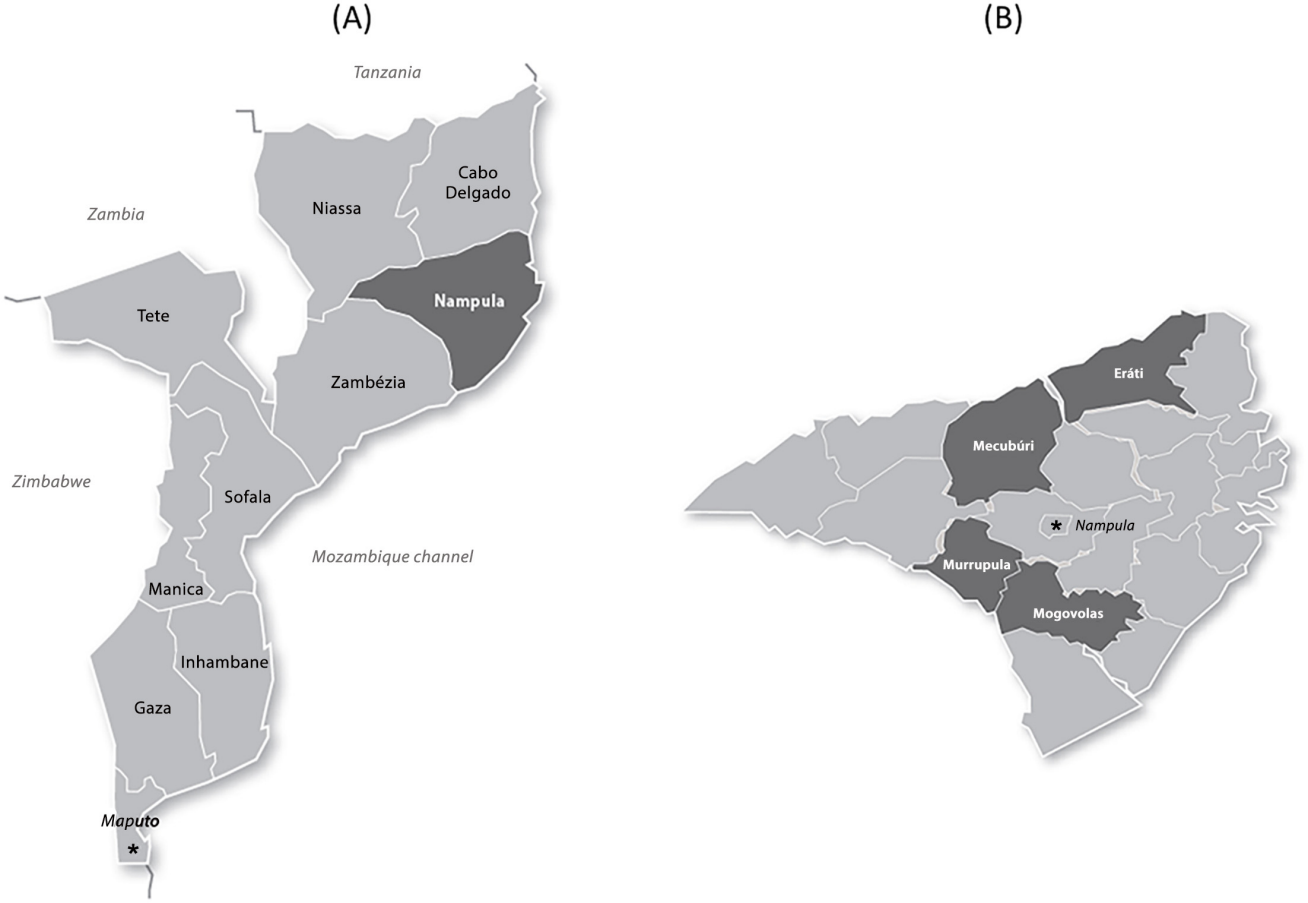
Maps of Mozambique and Nampula province. (A) Location of Nampula province within Mozambique highlighted dark grey. (B) Location of intervention districts within Nampula province highlighted dark grey.

According to the 2007 census, the total population of the four districts is approximately 708,000 [17]. Fig 1 shows the location of Nampula province within Mozambique, as well as the four intervention districts within the province.

In Mogovolas, MDA for schistosomiasis has been conducted annually since 2010. In Eráti, annual MDA campaigns started in 2012. In Mecubúri and Murrupula, the first MDA campaign for schistosomiasis was carried out in 2014. In all districts, the target for the MDA campaigns conducted prior to the KAP survey were school-age children between five and fourteen years of age. Campaigns conducted after the survey have targeted all persons over the age of five. Table 1 presents coverage figures for the MDA campaign preceding the survey as reported by the NTD Department at the Ministry of Health in Mozambique.

**Table 1:** MDA coverage rates in study districts for most recent MDA campaigns prior to the survey.

District	Year and month of campaign	Coverage rate
Mogovolas	May 2013	57%
Eráti	April 2014	84%
Mecubúri	April 2014	81%
Murrupula	April 2014	132%

Source: NTD Department, Ministry of Health, Republic of Mozambique.

Note that 2007 census data are used for the calculation of coverage rates. The census data are now widely believed to be inaccurate and out-of-date, which may explain coverage rates in excess of 100%. At the time of our survey, no MDA coverage surveys had been carried out to verify coverage figures in the region.

### Sampling

The survey was designed as a representative two-staged clustered cross-sectional household survey. The four study districts were considered as one sampling domain and all households were eligible for selection. The total sample size required was calculated in order to allow for an eventual comparison of proportions between baseline and endline. Determined by the need to give 80% power to detect a change of at least 10% between baseline and endline, it was estimated that a sample size of 389 households would be sufficient. This sample size allows baseline percentages to be estimated with a 95% confidence interval (CI) of width +/- 5%. In order to adjust for confounders, non-response and design effect, the sample size was doubled, resulting in a required sample size of 778 households. Sampling involved two stages:

1. From a list of the 68 enumeration areas in the four intervention areas, as defined by the 2007 Mozambique census, 40 enumeration areas were randomly selected as clusters using proportional-to-size methods.
2. In each selected cluster, 20 households were sampled using a simple random sampling approach. Thus, each household was equally likely to be selected. Where possible, existing lists of households were obtained from community leaders for this purpose. The sampling approach was adapted from the approach recommended for malaria indicator surveys [18]. For the purpose of the survey, a household was defined as a group of people who routinely live and eat together. One person per selected household was interviewed. The target individual for interviews was the person best placed to answer questions about the household’s health as nominated by the acting household head. Only household members over 18 years of age were eligible.

If no suitable respondent was available on the field researcher’s first visit to a household, field teams were instructed to make at least two additional attempts to interview an eligible household member at different times of the day. Households that could not be located, those where no eligible respondent was encountered despite at least three visits to the household and those where consent to be interviewed was not given were dropped from the sample without replacement.

### Data collectors

Field work was conducted over a period of two weeks in July 2014 by five field teams, each comprising four field researchers and one field supervisor. Field researchers were typically educated to university or college level, while field supervisors were expected to have completed a social sciences university degree. All field team members had previous experience of conducting or supervising field research and were native speakers of Macua, the language used in the study districts. Field researchers and supervisors attended a two-day training course conducted by the research supervisor and following a training guide developed by Malaria Consortium. The training covered data collection tools, field procedures and interview techniques. Field supervisors received an additional day’s training focusing on supervision of field teams as well as the sampling process. Pre-testing of the questionnaire and field procedures was done by the field researchers and supervisors who had attended the training. An additional half-day training session was conducted following the pre-test to discuss challenges identified and changes made as a result of the pre-test.

### Data collection tools

A structured questionnaire was developed in English and translated into Portuguese. Survey questions were subsequently translated into Macua. The accuracy of the translation was checked during the field researcher training and all questions and answer options were discussed in detail to ensure common understanding among the team members. Field supervisors were instructed to ascertain the most locally relevant term for schistosomiasis in the different localities in discussions with district health staff and community leaders, and to substitute this for the Macua term (‘ehiri’) provided on the questionnaire as required. Otherwise, field researchers were expected to read out questions exactly as they appeared on the questionnaire. All questions were designated as single or multiple-response and field researchers assigned one or more responses to the most suitable of a set of predefined answer options. If responses did not fit any of the predefined answer options, field researchers recorded them under ‘other’. Following questions about the drug that treats schistosomiasis, field researchers informed all respondents that the name of the drug is praziquantel and showed sample tablets of the drug before proceeding to ask whether anyone in the household had ever received the drug. Field supervisors were tasked with ensuring quality and consistency of the completed questionnaires and to seek clarification from field researchers as required.

### Data entry

Data were independently double-entered into EpiData 3.1 (EpiData Association) software. The two databases were then compared and, where differences between first and second entry were detected, data were verified by checking the record against the paper questionnaire.

### Data analysis

Data was further checked for consistency and prepared for analysis using STATA Version 12 (StataCorp LP). The survey procedures in STATA were used to account for the study design and potential clustering. All percentages reported are population average estimates. Responses recorded under ‘other’ by field researchers were reviewed and either re-assigned to an existing answer category, assigned to a newly created category or left in the ‘other’ category.

Associations were explored between a set of indicators relating to levels of KAP and different socio-demographic characteristics of respondents (sex, age group, education level and district), with statistical significance of the association tested using the chi-square test.

### Research ethics

The study was granted ethical approval by the University of Leeds School of Medicine Research Ethics Committee (SoMREC/13/071) and the Comité Nacional de Bioética para Saúde in Mozambique (42/CNBS/2014). Participation in the survey was voluntary, confidentiality and anonymity were guaranteed, and written informed consent was sought from all interviewees.

## Results

### Survey respondents

Of 800 households selected through the sampling process, 791 were interviewed. Five households were not interviewed because they could not be located or because no suitable interviewee was available. Four households were excluded from the analysis because the representative interviewed was below 18 years of age. None of the household representatives approached by the field researchers refused to be interviewed. Table 2 summarises survey respondents’ demographic characteristics and Table 3 shows respondents’ distribution by district.

**Table 2:** Survey respondents by sex, age and education (N = 791).

	N	%
**Sex**		
**Male**	402	51
**Female**	389	49
**Age**		
**18–35 years**	293	37
**36–55 years**	292	37
**>55 years**	83	11
**Don’t know**	120	15
**No answer**	3	0
**Education**		
**None**	193	24
**Primary**	507	64
**Secondary and higher**	81	10
**Don’t know**	4	1
**No answer**	6	1

**Table 3:** Survey respondents by district (N = 791).

	N	%
**District**		
**Mecubúri**	139	18
**Eráti**	274	35
**Murrupula**	119	15
**Mogovolas**	259	33

More women than men (difference 21%, 95% CI 15%-27%, p<0.01) had not had any formal education, whereas men were significantly more likely to achieve primary-level education than women (difference 20%, 95% CI 13%-27%, p<0.01). For most indicators, association with age was generally found not to be statistically significant. Where differences between districts were found to be statistically significant, there was no clear trend in terms of some districts showing consistently higher or lower levels of KAP compared with the other districts. In the following, only sex and education levels will be shown where the association with knowledge or practice indicators was statistically significant.

### Awareness of schistosomiasis

A large majority (91%, 95% CI 89%-93%) of respondents indicated that they had heard of a disease called schistosomiasis, using the local most common term for the disease (see Table 4). More men than women were aware of the disease (difference 6%, 95% CI 1.5%-10%, p<0.01). Differences in terms of education level were not found to be statistically significant.

**Table 4:** Respondents who were aware of schistosomiasis by sex and education level.

	N	%	95% CI
**Sex**			
**Male (N = 402)**	378	94	91–96
**Female (N = 389)**	293	88	85–91
**Education**			
**None (N = 193)**	168	87	81–92
**Primary (N = 507)**	471	93	91–95
**Secondary and higher (N = 81)**	73	90	81–95
**Don’t know (N = 4)**	4	100	100
**No answer (N = 6)**	5	83	33–98
**Total (N = 791)**	721	91	89–93

When those who were aware of the disease (N = 721) were asked where they had heard about schistosomiasis during the previous six months, 45% (95% CI: 38%-52%) stated that they had received information from a relative, neighbour or friend. Health professional/health facility (27%, 95% CI 21%-33%), community meeting (20%, 95% CI 14%-28%) and radio/TV (15%, 95% CI 11%-21%) were also frequently mentioned sources of information. About one third (31%, 95% CI 25%-37%) indicated that they had not heard about schistosomiasis during the previous six months. Note that multiple responses were possible for this question.

More than three quarters of those who were aware of the disease (N = 721) stated that they were concerned about schistosomiasis (83%, 95% CI 80%-87%) and thought that the disease can have long-term consequences for their personal health (78%, 95% CI 74%-82%). More than half (52%, 95% CI 46%-59%) indicated that their household is affected by schistosomiasis.

### Knowledge of risk behaviours

The majority (57%, 95% CI 51%-63%) of the respondents who were aware of schistosomiasis (N = 721) stated that they did not know how people become infected. Only 18% (95% CI 14%-22%) could name at least one correct risk behaviour (see Table 5). Correct knowledge of risk behaviours was more widespread among men than among women (difference 9%, 95% CI 5%-16%, p<0.01). Higher levels of education were also associated with better knowledge of risk behaviours (chi-square = 24.9, p<0.01).

**Table 5:** Respondents who named at least one correct risk behaviour^a^ by sex and education level.

	N	%	95% CI
**Sex**			
**Male (N = 378)**	87	23	19–27
**Female (N = 343)**	42	12	9–16
**Education**			
**No formal education (N = 168)**	17	10	6–16
**Primary (N = 471)**	83	18	14–22
**Secondary and higher (N = 73)**	27	37	27–48
**Don’t know (N = 4)**	1	25	3–78
**No answer (N = 5)**	1	19	2–72
**Total (N = 721)**	129	18	14–22

^a^ The following responses were considered correct risk behaviours: fetching contaminated water, fishing in infected water, poor hygiene/sanitation habits, bathing/swimming in the river, working in rice/agriculture fields.

The most frequently mentioned correct risk behaviours were fetching contaminated water (10%, 95% CI 7%-12%), poor hygiene/sanitation habits (8%, 95% CI 5%-11%) and bathing/swimming in the river (7%, 95% CI 5%-10%). Note that for the purpose of the analysis, responses relating specifically to drinking contaminated water were considered incorrect, as schistosomiasis is not thought to be acquired by ingesting the parasites. However, responses relating to fetching contaminated water were considered correct, as people are likely to come into contact with the parasite in the process of collecting drinking water.

**Fig 2:**
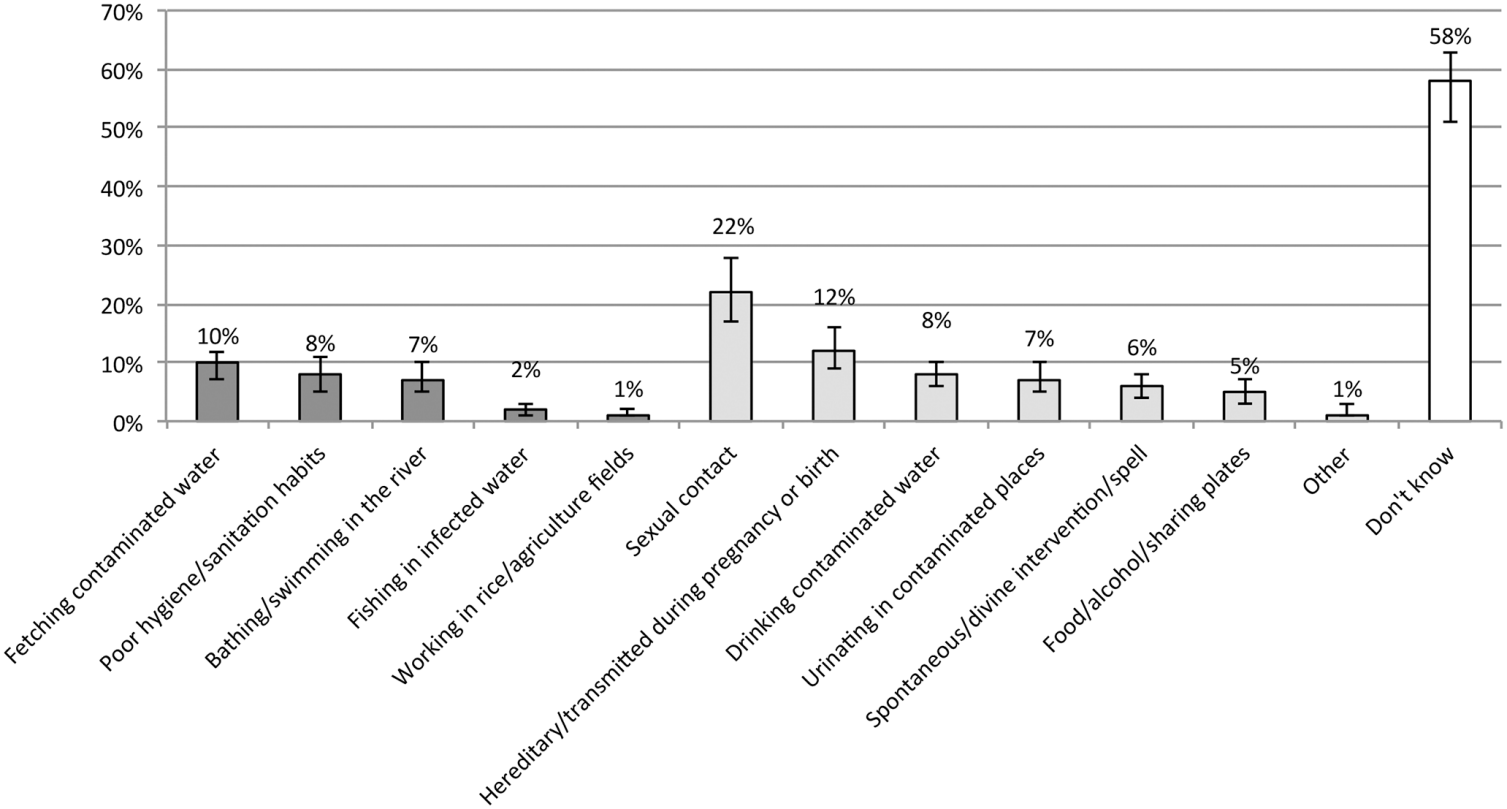
How do you get schistosomiasis? (N = 721). Range of responses: the first five options were considered correct responses (dark grey), the following seven were considered incorrect (light grey). 'Don't know' responses are shown on the far right (white). Bars indicate 95% CI for each data point.

Misconceptions with regard to how schistosomiasis is acquired were widespread: 22% (95% CI 17%-28%) thought the disease was acquired through sexual contact and 12% (95% CI 9%-16%) stated that it was hereditary or acquired during pregnancy or birth. Note that multiple responses were possible for this question and individual respondents may have mentioned both correct and incorrect risk behaviours. Fig 2 provides a breakdown of survey responses in terms of risk behaviours.

### Knowledge of transmission routes

When asked about how schistosomiasis is transmitted as opposed to how it is acquired, fewer than half (47%, 95% CI 42%-52%) of those who were aware of schistosomiasis (N = 721) indicated that an infected person can contribute towards spreading the disease. Of those who knew that schistosomiasis can be spread by infected persons (N = 338), only a quarter (26%, 95% CI 20%-32%) could name at least one transmission route correctly (see Table 6). While there was no significant difference in terms of correct knowledge of transmission routes between men and women, higher levels of education were associated with better knowledge (chi-square = 13.7, p<0.01).

**Table 6:** Respondents who could name at least one correct transmission route^a^ by sex and education.

	N	%	95% CI
**Sex**			
**Male (N = 186)**	48	26	19–34
**Female (N = 152)**	39	26	18–34
**Education**			
**None (N = 74)**	18	24	14–37
**Primary (N = 219)**	49	22	17–29
**Secondary and higher (N = 42)**	19	45	32–59
**Don’t know (N = 1)**	0	0	0
**No answer (N = 2)**	1	50	49–95
**Total (N = 338)**	87	26	20–32

^a^ The following responses were considered correct transmission routes: infected person urinating by water, infected person defecating by water.

About one quarter (24%, 95% CI 18%-30%) of those who knew that schistosomiasis can be spread by infected persons (N = 338) indicated that transmission can occur when an infected person urinates by water and one in ten (10%, 95% CI 7%-15%) stated that it occurs when an infected person defecates by water. The most frequently mentioned transmission route was a misconception relating to sexual behaviour, with the majority (81%, 95% CI 75%-86%) stating that the disease can be spread through unprotected sex. Multiple responses were possible for this question and individual respondents may have referred to both correct and incorrect transmission routes.

### Knowledge of prevention and treatment

Just over half (56%, 95% CI 51%-61%) of respondents who were aware of schistosomiasis (N = 721) said that they did not know how the disease can be prevented or treated. Only 13% (95% CI 10%-18%) could name at least two effective prevention or treatment mechanisms (see Table 7). Male respondents appeared to be more knowledgeable than female respondents (difference 7%, 95% CI 0%-20%, p<0.01). This also applied to respondents with higher educational achievements compared with those with lower achievements (chi-square = 12.7, p = 0.02).

**Table 7:** Respondents who could name at least two effective prevention or treatment mechanisms^a^ by sex and education level.

	N	%	95% CI
**Sex**			
**Male (N = 378)**	59	16	12–21
**Female (N = 343)**	32	9	6–14
**Education**			
**None (N = 168)**	18	11	6–18
**Primary (N = 471)**	55	12	9–16
**Secondary and higher (N = 73)**	17	23	15–35
**Don’t know (N = 4)**	0	0	0
**No answer (N = 5)**	1	20	2–72
**Total (N = 721)**	91	13	10–17

^a^ The following responses were considered effective prevention or treatment mechanisms: treat all people, treat all infected persons, treat the water source, protect the water source, avoid swimming, use well or pump water, build more latrines/observe better hygiene.

**Fig 3:**
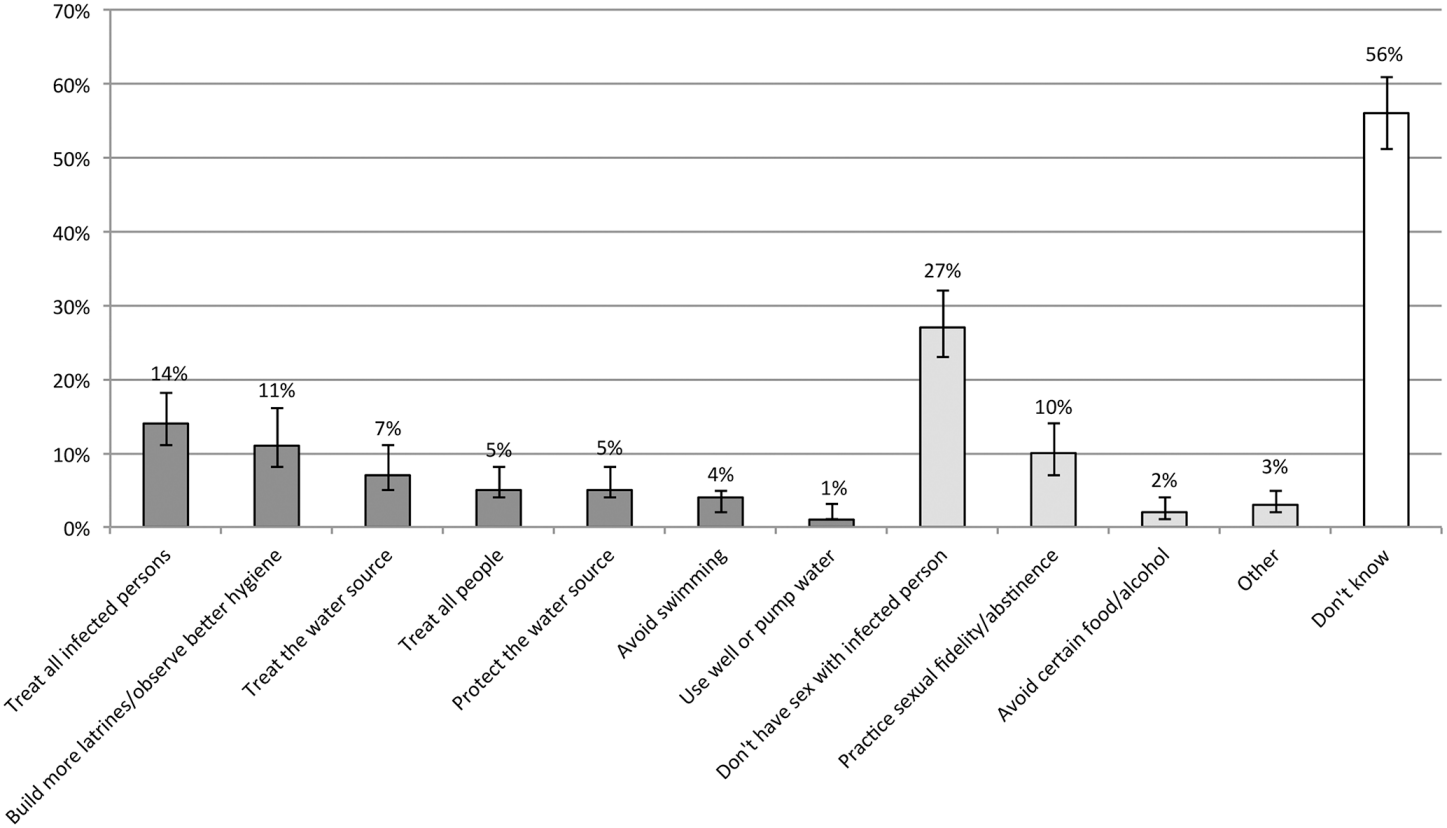
Do you know how you can avoid getting schistosomiasis? (N = 721). Range of responses: the first seven options were considered correct responses (dark grey), the following four were considered incorrect (light grey). 'Don't know' responses are shown on the far right (white). Bars indicate 95% CI for each data point.

The most frequently mentioned effective prevention or treatment mechanisms were treating infected people (14%, 95% CI 11%-18%), building more latrines/observing better hygiene (11%, 95% CI 8%-16%) and treating the water source (7%, 95% CI 4%-11%). Only 5% (95% CI 4%-8%) of respondents referred to MDA (‘treat all people’). Misconceptions relating to sexual contact were common, with 27% (95% CI 23%-32%) believing that schistosomiasis can be prevented by not having sex with an infected person and 10% (95% CI 7%-14%) stating that sexual fidelity/abstinence can prevent infections. Multiple responses were possible for this question and individual respondents may have given both correct and incorrect answers. Fig 3 shows responses relating to risk behaviours.

### Knowledge of symptoms and help-seeking

Of the respondents who were aware of schistosomiasis (N = 721), over two thirds (70%, 95% CI 66%-74%) could name at least two correct symptoms of the disease (see Table 8). Correct knowledge was more common among men than among women (difference 18%, 95% CI 10%-26%, p<0.01). Differences with regard to education level were not statistically significant.

**Table 8:** Respondents who could name at least two correct symptoms^a^ of schistosomiasis by sex and education level.

	N	%	95% CI
**Sex**			
**Male (N = 378)**	296	78	73–83
**Female (N = 343)**	208	60	55–67
**Education**			
**None (N = 168)**	106	63	56–71
**Primary (N = 471)**	334	71	66–76
**Secondary and higher (N = 73)**	57	79	67–87
**Don’t know (N = 4)**	3	75	22–97
**No answer (N = 5)**	4	80	26–78
**Total (N = 721)**	504	70	66–74

^a^ The following responses were considered correct symptoms: painful urination/painful bladder, frequent urination, blood in urine, blood in stool, fatigue, fever, headache, swollen stomach, diarrhoea, nausea/vomiting, rash/itch, weight loss, back ache, stomach ache, genital pain.

The symptoms most frequently mentioned were blood in urine (72%, 95% CI 67%-77%), painful urination/pain in bladder (72%, 95% CI 67%-77%), weight loss (36%, 95% CI 28%-46%), frequent urination (31%, 95% CI 26%-36%) and rash/itch (17%, 95% CI 13%-22%). Many respondents specified that the rash or itch affects the urogenital area. All of these are correct symptoms of schistosomiasis infection, although some of them are non-specific and may relate to other conditions. Incorrect symptoms such as swollen legs or paralysis were mentioned very infrequently.

A large majority (93%, 95% CI 90%-96%) of those respondents who knew at least one correct symptom of schistosomiasis (N = 584) stated that they would seek help if they had symptoms of the disease. Of those who would seek help (N = 544), 84% (95% CI 78%-88%) would visit a health facility or consult a health worker. Traditional healers (39%, 95% CI 33%-45%) and pharmacies or drug vendors (16%, 95% CI 12%-23%) were also frequently mentioned. The most frequent reasons given by the 19 respondents who indicated they would not seek help if they had symptoms of schistosomiasis were: ‘I don’t know who to consult’ (n = 12), ‘I have no money’ (n = 10), ‘I never seek medical help’ (n = 8) and ‘I’m ashamed’ (n = 3). Multiple responses were possible for all questions presented in this section.

### Praziquantel and MDA

Of those respondents who were aware of schistosomiasis (N = 721), more than half (61%, 95% CI 55%-67%) indicated that they did not know whether there was a drug that treats the disease and 8% (95% CI 6%-11%) said that such a drug does not exist. 28% (95% CI 23%-34%) stated that there was a drug. Only 3% (95% CI 2%-4%) correctly named praziquantel when prompted for its name.

Only a small minority (9%, 95% CI 7%-13%) of respondents from households with at least one child under the age of 18 (N = 579) reported that at least one of the children in the household had ever received praziquantel. Of the 88% (95% CI 84%-91%) who reported that their children had never received the drug (N = 507), a large majority (92%, 95% CI 89%-95%) indicated that they would want their children to take the medication if offered through MDA. Among the 27 respondents who indicated they would not want their children to take praziquantel, again or for the first time, the majority (n = 24) stated that children should only take medication if they are ill.

### Protective practices

Of the respondents who were aware of schistosomiasis (N = 721), just under half (46%, 95% CI 41%-50%) reported that they do nothing to protect themselves and their household from the disease. Among these individuals (N = 330), 78% (95% CI 72%-83%) explained that that they did not know what they can do and 12% (95% CI 8%-20%) indicated that they did not have enough money to practice any protective behaviours. Only about one third of respondents (32%, 95% CI 27%-38%) stated that they did something to protect themselves and their household from schistosomiasis.

However, only 39% (95% CI 31%-48%) of those respondents who stated that they did something (N = 230) cited at least one effective protective behaviour when asked to specify what they did (see Table 9). Practice of effective behaviours was more common among respondents with higher education levels (chi-square = 17.2, p<0.01). Differences in terms of sex were not statistically significant.

**Table 9:** Respondents who practiced at least one effective protective behaviour^a^ by sex and education level.

	N	%	95% CI
**Sex**			
**Male (N = 133)**	57	43	34–53
**Female (N = 97)**	32	33	21–48
**Education**			
**None (N = 46)**	11	24	13–39
**Primary (N = 150)**	56	38	29–47
**Secondary and higher (N = 32)**	22	69	52–83
**Don’t know (N = 0)**	0	0	0
**No answer (N = 2)**	0	0	0
**Total (N = 230)**	89	39	31–48

^a^ The following responses were considered effective behaviours: avoid swimming or wading in contaminated water, boil bathing water, use latrine.

**Fig 4:**
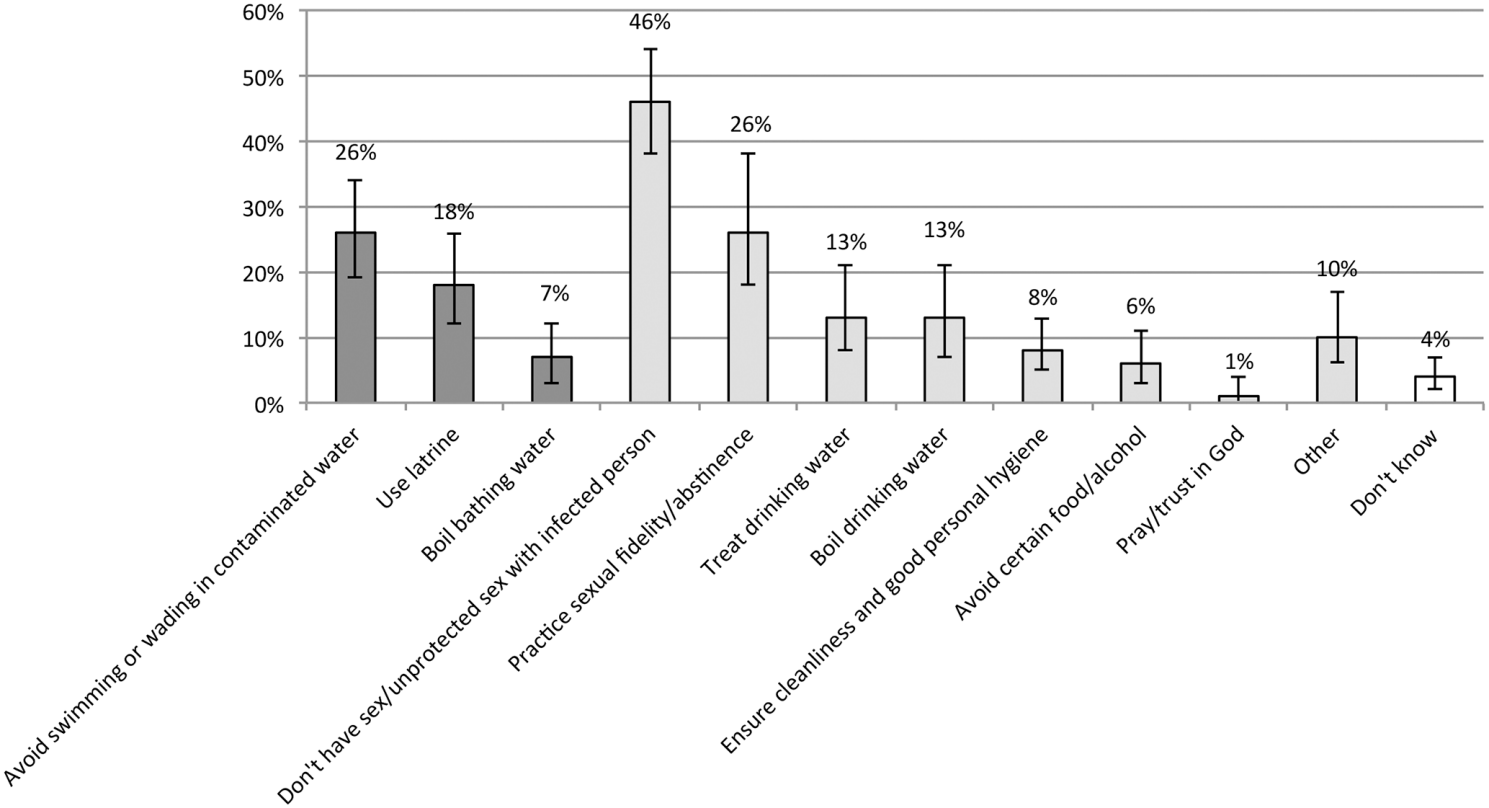
What do you do to protect you and your household from schistosomiasis? (N = 230). Range of responses: the first three options were considered correct responses (dark grey), the following eight were considered incorrect (light grey). 'Don't know' responses are shown on the far right (white). Error bars indicate 95% CI for each data point.

Of the respondents who were aware of schistosomiasis and stated that they do something to protect themselves (N = 230), 26% (95% CI 12%-26%) mentioned that they avoid swimming/wading in contaminated water and 17% (95% CI 12%-26%) said they use latrines. Misconceptions relating to sexual behaviour were common, with about half of respondents (46%, 95% CI 38%-54%) stating they do not have unprotected sex with an infected person and 26% (95% CI 18%-38%) indicating that they practice sexual fidelity or abstinence. Treating (13%, 95% CI 8%-21%) and boiling (13%, 95% CI 7%-21%) drinking water were also frequently mentioned. For the purpose of the analysis, responses relating to drinking water were considered ineffective behaviours. Multiple responses were possible for this question and individual respondents may have cited both effective and ineffective behaviours. Fig 4 details respondents’ answers with regard to the protective behaviours they practice.

## Discussion

While over 90% of respondents had heard of schistosomiasis and the majority of respondents saw the disease as a matter of concern that can have serious health consequences, correct knowledge of the disease was low. This is in line with findings from a recent qualitative study in Kenya [19]. Misconceptions are widespread. The most frequently mentioned risk behaviours, transmission routes and prevention and treatment mechanisms often relate to the belief that schistosomiasis is transmitted through sexual contact. Other misconceptions relate to the belief that schistosomiasis is a hereditary disease or that it is acquired by eating certain types of food. Similar misconceptions have been reported in other studies in various African countries [20–23]. Correct knowledge of schistosomiasis was particularly low with regard to transmission routes, with just over 10% of those who were generally aware of the disease citing one correct route. Moreover, it was evident that many respondents did not distinguish between risk behaviours, i.e. how schistosomiasis is acquired, and transmission routes, i.e. how it is passed on. Given the high prominence of MDA in schistosomiasis prevention and control strategies both internationally and nationally, it was also worrying to find that knowledge of MDA and knowledge of praziquantel as a drug that treats schistosomiasis was very low, with only 5% of respondents citing MDA as a prevention mechanism and 61% stating that they did not know whether a drug existed to treat the disease. The findings highlight a substantial knowledge gap among the population, despite communities’ exposure to MDA campaigns in the very recent past: in three out of the four districts, an MDA campaign had been conducted just two months prior to the survey. There was no discernible pattern in terms of levels of KAP between districts, even though in one district annual MDA started as early as 2010, whereas in other districts MDA only started in the year of the survey.

Very few respondents reported that their children had ever received praziquantel. This is surprising and concerning given that previous MDA campaigns in the region have targeted school children and drug distribution was mainly through schools. However, it is plausible that because of the distribution mechanism, caregivers may not have been aware of the campaigns and may not know whether their child received treatment. In an environment where childcare is typically the responsibility of female household members, this may apply to male survey respondents in particular. There was no statistically significant difference between districts with regard to respondents who reported that their children had ever received praziquantel, despite different levels of MDA coverage reported from the four study districts from previous MDA campaigns, casting further doubt on the accuracy and reliability of available coverage data.

A range of challenges with regard to conducting MDA campaigns have been reported anecdotally by Ministry of Health and implementation partners staff, which may, in part, account for the lower than expected levels of knowledge and reported exposure to MDA:

- Capacity and planning challenges have resulted in irregular delivery of MDA, resulting in varying coverage levels. Some communities might not have benefited from MDA.
- MDA has been delivered to varying target groups (adults, school-age children) for various diseases in addition to schistosomiasis. Treatment for schistosomiasis has been provided either in combination with treatment for lymphatic filariasis or soil-transmitted helminthiases. This may have led to confusion among the population with regard to the purpose of the campaigns.
- There have been recurrent reports of localised antagonism towards health authorities and providers among some communities in the study area, sometimes linked to the outbreak of diseases such as cholera. Though this survey suggests that acceptability of MDA is high, it cannot be ruled out that feelings of distrust persist, which may have prevented some communities from engaging with MDA campaigns.
- The capacity for implementing comprehensive and intensive social mobilisation about NTDs has typically been limited to just before and during MDA campaigns, which may have resulted in some people not being exposed to health education messages.
- Social mobilisation may have been restricted to schools, so knowledge may not have filtered through to adult caregivers.

In contrast to knowledge of risk behaviours, transmission routes and prevention and treatment mechanisms, knowledge of the symptoms of schistosomiasis was comparatively high. This may be a result of the fact that the disease can have a multitude of symptoms, most of which are non-specific. The most widely mentioned symptoms generally related to the urogenital area, which may explain why misconceptions with regard to schistosomiasis as a sexually transmitted disease are so widespread.

The vast majority of respondents indicated that they would seek help if they experienced symptoms of the disease. While many people would seek help from a health worker or at a health facility, a significant number would consult a traditional healer or potentially an informal drug vendor, indicating that it will be important to engage with these traditional or private providers of care in order to improve access to adequate treatment. Preference for treatment from traditional healers was also reported from a study in Tanzania [20]. Encouragingly, acceptability of MDA among the population appears high with around 90% stating that they would want to receive praziquantel for themselves or their children if offered during an MDA campaign. This appears to contradict reports in the literature of widespread fear of treatment through MDA and opposition to the distribution mechanisms as barriers to MDA uptake in Uganda [9]. Only a small minority of survey respondents expressed similar concerns.

In line with the generally low levels of correct knowledge of risk behaviours, transmission routes and disease prevention and treatment, levels of adoption of effective protective behaviours was low, confirming findings from studies conducted in Ghana and Côte d’Ivoire [21, 22]. Many of those who indicated that they do practice protective behaviours referred to avoiding unprotected sexual contact, which is ineffective against schistosomiasis. Over 75% of respondents who stated that they do nothing indicated that they do not know what they can do to protect themselves and their families from the disease, highlighting the need to improve knowledge about how to prevent schistosomiasis among the population. There was also a significant number of respondents who implied that their personal circumstances did not allow them to take action, a barrier that has also been reported from communities around Lake Victoria in Uganda and Kenya [19, 24], suggesting that recommended protective behaviours need to take into account the local context. It will also be important to strengthen communities’ capacity to take locally relevant communal action.

Higher education levels were fairly consistently associated with higher levels of KAP. Men were also consistently found to be more knowledgeable than women. This may be attributed to the fact that, due to their work in agriculture or fishing, men may be more exposed to the parasite and the disease, and therefore may receive treatment or advice more frequently than women. Men’s higher levels of knowledge may also be associated with the fact that men tend to receive more formal education and, as shown, formal education levels are linked to better knowledge of the disease.

### Limitations

The questionnaire for this study was originally developed in English and subsequently translated into Portuguese and then Macua. Accuracy of the Macua translation was discussed during the field researcher training to ensure consistent interpretation of the questions among the field team, but some subtleties may have been missed in the translation process. In addition, there is no tradition of reading and writing in Macua and field researchers, though native speakers of the language, required considerable practice and training on reading out the questionnaire in the local language.

While qualitative formative research carried out in preparation for the community dialogues study found that local understandings of schistosomiasis are largely congruent with the biomedical model and field teams took care to establish the correct local term to be used during the interview for each community in the sample, it cannot be ruled out that some respondents may have referred to disparate disease concepts. It should also be noted that while respondents were shown sample praziquantel tablets, they might not have recognised the drug or might not have accurately remembered whether or not their children had previously taken the medication.

Allowing households to nominate the interviewee may have introduced an element of bias, as the person best placed to answer questions about the household’s health is also likely to be more aware and knowledgeable of health issues than other household members. This means estimates presented are likely to be overestimates of the population of individuals. Similarly, desirability bias may have led some respondents to give answers they felt were socially acceptable.

Note also that the survey was designed as a baseline KAP survey for a community engagement intervention aimed at adults, which encourages participants to participate in MDA campaigns as one of a number of recommended behaviours to improve schistosomiasis prevention and control. However, the intervention does not involve distributing drugs. Consequently, the survey, while interested in people’s previous exposure to MDA, was not designed as a coverage survey. It did not ask about specific MDA campaigns and it purposively excluded children as direct respondents. Findings with regard to exposure to MDA can therefore not be used to conclusively assess MDA coverage in the region.

### Conclusion

This study adds to the limited literature exploring KAP of schistosomiasis prevention and control at the community level. In the light of high schistosomiasis prevalence rates in Mozambique, its findings can inform policy and practice in particular with regard to social mobilisation surrounding MDA campaigns and improving the adoption of recommended protective behaviours more generally. It will be essential to educate communities about the existence of a drug to treat schistosomiasis and its safety and efficacy, as well as the rationale behind MDA, in order to capitalise on the high acceptability of MDA and to help achieve and maintain high MDA uptake. Given people’s readiness to seek help from qualified providers, it will also be important to ensure availability of drugs, diagnostic equipment and up-to-date treatment guidelines, so health facilities can meet the demand that social mobilisation may create.

As our results are similar and complementary to findings from (often small-scale or qualitative) studies conducted in other African countries, they should be of relevance to the wider African context. Low levels of knowledge of the causes of schistosomiasis and how to prevent the disease, coupled with persisting misconceptions and a tendency to rely on traditional cures continue to pose barriers to effective disease prevention and control. Efforts should be made to improve adequate knowledge at the individual and community level, in particular among women and in areas where education levels are generally low. As the adoption of effective protective behaviours depends on circumstances as well as knowledge, communities need to be encouraged to take ownership of schistosomiasis prevention and control, such as improving sanitation behaviours, and to participate in decisions with regard to identifying relevant and appropriate actions to overcome local challenges. Community dialogues could be one approach that may improve knowledge and strengthen the adoption of protective behaviours at the community level.
